# Ventilation of horse riding helmets: what is the connection between laboratory and field measurements?

**DOI:** 10.1186/2046-7648-4-S1-A87

**Published:** 2015-09-14

**Authors:** Matthieu Jolly, Alexia Cariou, Emmanuelle Koralewski

**Affiliations:** 1 Thermal Affective and Laboratories, Decathlon SportsLab, Villeneuve d'Ascq, France; 2 Fouganza, equestrian brand of Decathlon, Villeneuve d'Ascq, France

## Introduction

Helmets have received little attention in the literature[1] concerning thermal comfort. Whether it is for motorcycling or cycling, ventilation of helmets has become an issue [2,3]. Relationships between heat loss and the effects perceived vary among helmets. Fouganza, the equestrian brand of Decathlon, has made ventilation of horse riding helmets a priority, first laboratory and then field measurements were performed in order to evaluate the validity of laboratory measures and also to rank a range of helmets on a scale from 1 to 5.

## Methods

The determination of thermal and evaporative resistance was assessed on seven helmets, using a head manikin in a climatic chamber under 20°C, 40 % rh and two wind speeds (1,3 and 15,0 km.h^-1^). Four indicators (Rc_low speed_, Rc_high speed _, Re_low speed _, Re_high speed_) were thus obtained.

In order to better understand the behaviour of the helmets during field tests, a specific questionnaire was created for 15 horse riders (from Gallop 3 to Gallop 7) to collect the subjective responses of four helmets, on a 9-points-scale (from not ventilated to very ventilated), after an intense exercise of 45 minutes undertaken at the Equestrian Centre of Roubaix.

## Results

From the four indicators, Rc_low speed _was found to be significantly correlated with ventilation of horse riding helmets. The general grading remained the same by selecting the other indicators Re_high speed _, Re_low speed _and Rc_high speed_, but they are not more representative of the real use.

Thus we obtained the coefficients of the equation *ventilation = α.Rc_low speed _+ β*, allowing us to rank our range of helmets.

## Discussion and conclusion

The 0,179 m².K.W^-1 ^range in the results can be explained by the no ventilation helmet (C400) compared to the ventilation oriented conception (Samshield, with channels, a way in and way out for the wind). The field results have highlighted the best choice for our indicator of ventilation, but with only four helmets tested in the field. In the future, results could be adjusted depending on the wind speed and the selection of head segments for the calculation. Brühwiler (2004) also showed that the inclination would be another area of investigation^3^. The test method will also lead to strong improvement in the conception of helmets regarding ventilation.

**Figure 1:**
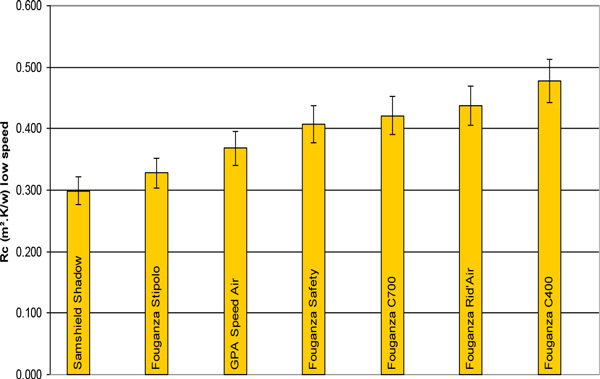


## References

[B1] Taylor NA Caldwell JN Dyer R The physiological demands of horseback mustering when wearing an equestrian helmet Eur J Appl Physiol 2008 104 2 289 296 10.1007/s00421-007-0659-5 18176814

[B2] Bogerd CP Rossi RM Brüwiler PA Thermal perception of ventilation changes in full-face motorcycle helmet_subject and manikin study Ann Occup Hyg 2011 55 2 192 201 10.1093/annhyg/meq074 20959389

[B3] Brühwiler PA Ducas C Huber R Bishop PA Bicycle helmet ventilation and comfort angle dependence Eur J Appl Physiol 2004 92 6 698 701 10.1007/s00421-004-1114-5 15138828

